# Multiple Environmental Determinants of *Opuntia stricta* Distribution: Habitat Suitability Across Global Biomes

**DOI:** 10.3390/biology15181635

**Published:** 2026-09-16

**Authors:** Anil Poudel, Pradeep Adhikari, Prabhat Adhikari, Yong Ho Lee, Sun Hee Hong

**Affiliations:** 1 Department of Plant Resources and Landscape Architecture, College of Agriculture and Life Sciences, Hankyong National University, Anseong 17579, Republic of Korea; aneeelily@gmail.com (A.P.); adprabhat2051@gmail.com (P.A.); 2 Institute of Humanities and Ecology Consensus Resilience Lab, Hankyong National University, Anseong 17579, Republic of Korea; pdp2042@gmail.com

**Keywords:** biomes, environmental variables, habitat suitability, invasive species, niche overlap, *Opuntia stricta*

## Abstract

*Opuntia stricta* is a highly invasive cactus species that threatens arid and semiarid ecosystems worldwide. We identified the optimal species distribution model (SDM) to predict its global habitat suitability using three hierarchical frameworks: climate only, climate with soil, and climate with soil and human influences. Among five modeling algorithms, Maximum entropy (MaxEnt) showed the highest predictive performance, supporting its use as the primary habitat-suitability model. Habitat suitability was driven mainly by climatic variables, with precipitation of the driest month, isothermality, and potential evapotranspiration contributing 30.7%, 26.7%, and 18.3%, respectively, whereas soil and human factors made smaller contributions. Biome-level analysis revealed that Mediterranean ecosystems exhibited the highest proportional habitat suitability (48.5%). The climate-only model predicted 7.35% of global land as suitable, which decreased by 34% after soil and human variables were incorporated. Native-introduced niche-overlap analysis further showed that additional environmental predictors refined environmental niche characterization across modeling scenarios. These findings highlight the importance of integrating multiple environmental predictors to improve habitat-suitability predictions and provide a robust framework for supporting the management of *O. stricta* and other invasive plant species worldwide.

## Simple Summary

*Opuntia stricta* is a globally invasive cactus that threatens many natural ecosystems. In this study, we assessed the temporal patterns of its reported occurrence records and predicted its potential distribution under current environmental conditions. Our results indicate a marked increase in reported occurrences in recent decades and show that habitat suitability is primarily influenced by climatic factors, particularly temperature and moisture availability. Mediterranean and subtropical regions showed relatively high predicted habitat suitability for *O. stricta*. Our comparison of native and introduced environmental niches also provided new insights into the environmental conditions associated with the global spread of *O. stricta.* Soil predictors provided secondary predictive information, whereas the HII provided comparatively little additional information. The continued spread of *O. stricta* may seriously impact agriculture, biodiversity, and ecosystem functioning. Therefore, improved monitoring, early detection, and careful management are essential to prevent further expansion and reduce future risks. This study provides important information that can guide the development of effective prevention and control strategies worldwide.

## 1. Introduction

Invasive alien plant species (IAPs) represent among the most significant threats to global biodiversity, ecosystem function, and socioecological stability [1,2]. Their broad environmental tolerance, rapid growth, and ability to exploit disturbed habitats enable them to establish and spread beyond their introduced ranges, where they can alter native plant communities, disturbance regimes, nutrient cycling, and ecosystem resilience [1,3]. The establishment and distribution of IAPs are influenced by interacting climatic, edaphic, and anthropogenic factors that determine whether introduced populations can persist under local environmental conditions [3,4]. Understanding these environmental relationships is therefore important for identifying potentially suitable areas and supporting effective monitoring and management strategies.

Climate is a major determinant of plant distribution because temperature, precipitation, and water availability directly influence physiological performance, growth, reproduction, and survival [5]. Elevated temperatures, shifting precipitation patterns, and increased atmospheric CO_2_ concentrations directly increase the physiological efficiency, growth trajectories, and reproductive success of numerous IAPs, providing them with a competitive advantage over native flora [6]. Additionally, milder winters, extended growing seasons, and climate-induced disturbances, such as droughts and wildfires, create new opportunities for invaders to colonize habitats that were previously unsuitable [7,8]. These environmental transformations can simultaneously weaken native plant communities, especially in ecosystems where water availability and temperature limit vegetation productivity, thereby lowering natural resistance to invasion and establishing conditions that favor the establishment and spread of IAPs [9].

Human activities also strongly influence the introduction and dispersal of invasive plants [1,10]. Important anthropogenic pathways include intentional cultivation for ornamental or utilitarian purposes, livestock-mediated propagule transport, and unintentional dispersal along roads, settlements, and other transportation networks [11,12]. These processes may facilitate establishment in new regions and increase propagule pressure. Nevertheless, broad indicators of human influence may capture only part of these processes, particularly because introduction and dispersal pathways can differ from the environmental conditions that determine local habitat suitability.

Edaphic conditions provide an additional environmental filter influencing plant establishment and persistence. Soil properties such as texture, pH, nutrient availability, organic matter content, bulk density, and water-holding capacity can affect germination, rooting, nutrient acquisition, and competitive interactions between invasive and native vegetation [2,13,14,15]. Incorporating soil predictors alongside climatic variables can therefore improve characterization of environmental habitat suitability by accounting for conditions that may not be represented by climate alone.

*Opuntia stricta*, commonly known as erect prickly pear, is a perennial cactus native to the Americas, particularly the southeastern United States, Mexico, and the Caribbean [16,17]. During the past two centuries, the species has been introduced widely into Africa, Australia, Asia, and the Mediterranean region, primarily for ornamental purposes, live fencing, and other utilitarian uses [16,17]. Following introduction, *O. stricta* has become naturalized or invasive in numerous rangelands, agricultural areas, and protected ecosystems worldwide [18,19]. Severe invasions have been documented in Australia, southern and eastern Africa, India, Madagascar, and parts of the Mediterranean Basin [16,20], although its occurrence and impacts may remain underreported in some regions. Land-use change, overgrazing, deforestation, and other disturbances can facilitate establishment and persistence of the species in degraded environments [18,20,21].

The invasive success of *O. stricta* is supported by several biological and physiological characteristics. The species reproduces both sexually through seeds and vegetatively through detached cladodes that can readily root and establish new plants [16,22]. Its fleshy fruits are consumed by birds and mammals, facilitating seed dispersal, while detached cladodes may also be transported by animals, floodwaters, and human activities [16,22,23]. In addition, *O. stricta* uses crassulacean acid metabolism (CAM), which allows nocturnal gas exchange and reduces water loss under hot and dry conditions [24,25]. Together with tolerance to drought, variable rainfall, nutrient-poor soils, and other environmental stresses, these traits contribute to the ability of *Opuntia* species to persist in arid and semiarid environments [26,27]. *O. stricta* is consequently recognized among the world’s 100 worst invasive species [28].

The ecological and socioeconomic impacts of *O. stricta* are substantial. Dense infestations can replace native vegetation, reduce habitat quality, restrict livestock and human movement, and diminish the productivity of rangelands and agricultural systems. In Kenya, extensive infestations have been associated with reduced grazing capacity, livestock health problems, and declines in native vegetation [26]. The species also threatens biodiversity in protected areas such as Kruger National Park in South Africa [29]. Historically, *Opuntia* invasions affected millions of hectares of pastoral land in Australia before successful biological-control programs substantially reduced infestations [30]. In Madagascar and eastern Africa, *O. stricta* has similarly affected livestock production, fodder availability, human access, and rural livelihoods [26,31]. These impacts underscore the need to identify environmentally suitable areas where monitoring and management may be especially important.

Species distribution models (SDMs) provide a useful framework for estimating habitat suitability by relating species occurrence records to environmental predictors [32]. A variety of statistical and machine learning approaches, including artificial neural networks (ANNs), random forests (RFs), generalized additive models (GAMs), boosted regression trees (BRTs), and maximum entropy modeling (MaxEnt), have been widely applied in ecological distribution studies. MaxEnt is particularly useful for presence-background data and has been extensively applied to invasive species because it can accommodate nonlinear relationships among occurrence records and environmental variables [33,34]. Previous studies have also demonstrated the utility of SDMs for identifying environmentally suitable areas for invasive cactus species and supporting spatially targeted management [35,36].

Despite the widespread occurrence and ecological impacts of *O. stricta*, a global assessment integrating climatic, edaphic, and anthropogenic predictors, together with an explicit comparison of native and introduced environmental niches, remains valuable for understanding the environmental conditions associated with its current distribution. Because occurrence records from both the native and introduced ranges were pooled for the global SDMs, the resulting predictions are interpreted as estimates of environmental habitat suitability across the species’ currently occupied global environmental range rather than as formal estimates of invasion probability outside the native range. Potential differences between native and introduced environmental niches were therefore evaluated separately using niche-overlap and niche-dynamics analyses.

Accordingly, this study aims to: (1) characterize temporal and geographic patterns in reported occurrence records of *O. stricta*; (2) identify the climatic, edaphic, and anthropogenic predictors most strongly associated with its global habitat suitability; (3) compare global habitat-suitability predictions under climate-only, climate + soil, and climate + soil + human-influence frameworks; (4) characterize habitat suitability across major global biomes; and (5) assess native-introduced niche overlap, stability, expansion, and unfilling. By integrating temporal occurrence patterns, multivariable habitat-suitability modeling, and native-introduced niche analysis, this study provides a current environmental baseline that can support monitoring, early detection, and management planning for *O. stricta*.

## 2. Materials and Methods

### 2.1. Occurrence Data

**Figure 1:**
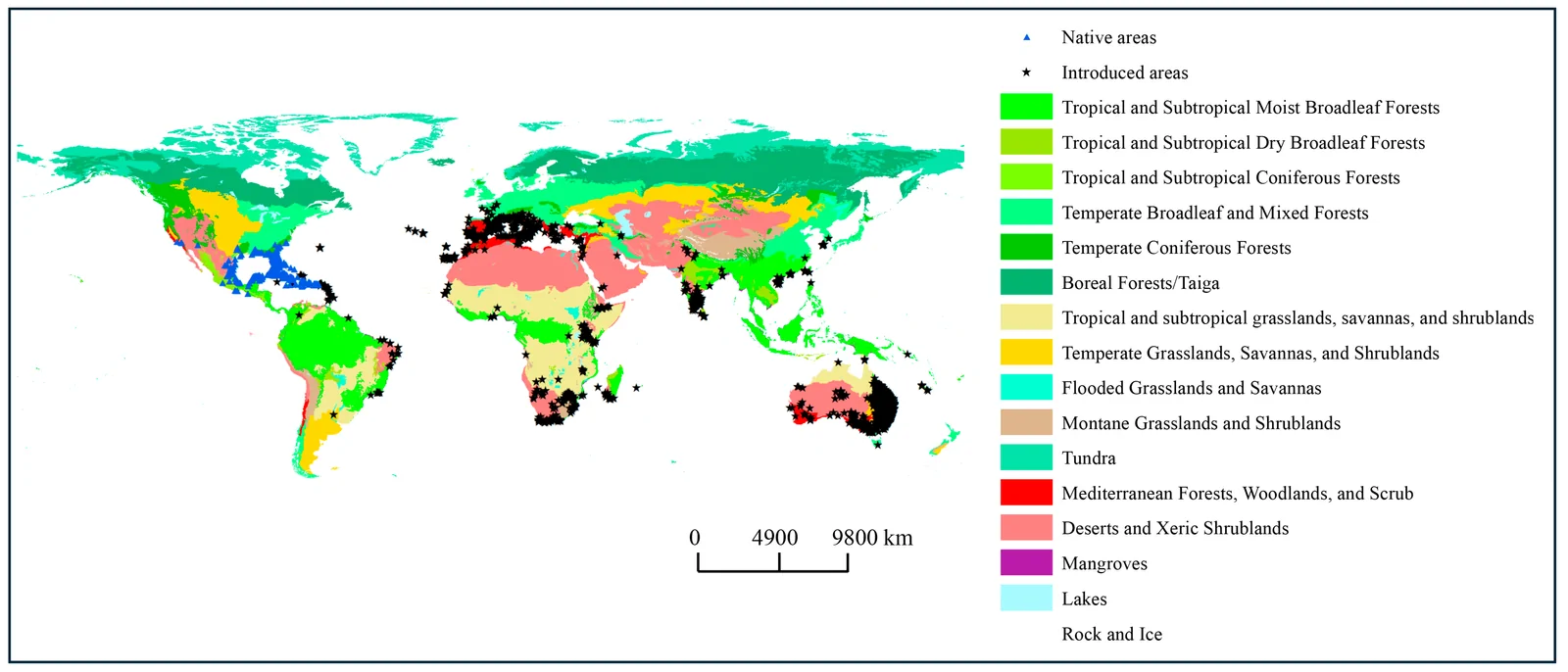
Worldwide distribution of major terrestrial biomes overlaid with native and introduced occurrence points of *O. stricta*. The map illustrates the broad ecological zones in which the species is present globally.

A total of 26,196 occurrence records of *O. stricta* were retrieved from the Global Biodiversity Information Facility (GBIF occurrence download. Available online: https://www.gbif.org/taxon/6SRQ7; accessed on 21 June 2025). To improve data quality, duplicate, erroneous, and taxonomically mismatched records were carefully screened and removed. To minimize spatial autocorrelation and sampling bias, the occurrence points were spatially filtered using a 2.5 arc-minute resolution grid cell with the “Spatially Rarefy Occurrence Data” tool in ArcGIS SDM Toolbox v.2.4 [7,37,38,39,40,41]. Occurrence records were classified into native- and introduced-range groups based on the documented native distribution of *O. stricta* in the southeastern United States, Mexico, and the Caribbean [16,17,18]. Records occurring within the documented native geographic range were classified as native-range occurrences, whereas records outside this range, in regions where the species has been introduced, were classified as introduced-range occurrences. After spatial rarefaction, a total of 3326 unique occurrence records remained, of which 573 were native-range records and 2753 were introduced-range records. These records were subsequently used for species distribution modeling (Figure 1).

### 2.2. Environmental Data

The environmental variables for *O. stricta* distribution modeling were compiled from multiple global databases that represent climatic, edaphic, and anthropogenic factors (Table S1). Current climatic data spanning 1973–2013 were obtained from the PaleoClim v1.2 dataset at a spatial resolution of 2.5 arc minutes (http://www.paleoclim.org, accessed on 15 January 2025) [42], including 19 bioclimatic variables representing annual trends, seasonality, and extreme climatic conditions at a spatial resolution of 2.5 arc minutes (~4.5 km^2^) [42,43]. Additionally, climatic variables, such as potential evapotranspiration (PET) and the aridity index (AI), which reflect water availability and atmospheric moisture demand, were incorporated as inputs for the species distribution model. These data were downloaded from the CGIAR-CSI database (http://www.csidotinfo.wordpress.com, accessed on 5 May 2025) [44,45].

Ten soil variables were obtained from the ISRIC-World Soil Information database (SoilGrids250m, version 2.0) via the SoilGrids web service (https://rest.isric.org/soilgrids/v2.0; accessed on 27 March 2019) at depths of 0–200 cm and a spatial resolution of 30 arc-seconds (~1 km^2^) [46]. The selected variables included soil organic carbon content (ORCDRC, g kg^−1^), pH in H_2_O (PHIHOX ×10), bulk density (BLDFIE, g cm^−3^), cation exchange capacity (CEC, cmol kg^−1^), sand content (SNDPPT, %), silt content (SLTPPT, %), clay content (CLYPPT, %), coarse fragments (CRFVOL, %), available water capacity (AWch), and soil texture class (TEXMHT).

To account for anthropogenic impacts on habitat suitability, the global human influence index (HII) was incorporated at a spatial resolution of 1 km^2^, providing a comprehensive measure of human influence on terrestrial ecosystems by integrating data on human population density, land use, accessibility, and electrical power infrastructure [47,48]. Additionally, terrestrial biomes/ecoregions worldwide were included to characterize broad ecological zones. These data were downloaded from the World Wildlife Fund–US (WWF) and are based on the Terrestrial Ecoregions of the World classification [49]. All environmental layers were resampled to a common spatial resolution of 2.5 arc-minutes to ensure consistency across all predictor variables, with data processing conducted using R statistical software v.4.3.3 (http://www.r-project.org, accessed on 6 June 2026) with the raster and sp packages.

To reduce multicollinearity among environmental predictors, Pearson’s correlation coefficients were calculated among raster values across the global terrestrial study extent using the Band Collection Statistics tool in ArcGIS 10.8 (Esri, Redlands, CA, USA). Predictor pairs with an absolute correlation coefficient greater than 0.75 (|r| > 0.75) were considered highly correlated. When two variables exceeded this threshold, one predictor was retained primarily on the basis of its ecological relevance to *O. stricta*, biological interpretability, and representation of a distinct environmental process [38,50,51] (Table S2). Preliminary model outputs were used only as a secondary diagnostic and were not used as the primary criterion for predictor elimination. Correlation screening was conducted once using the complete candidate predictor set, after which the retained variables were assigned to the appropriate climate-only, climate + soil, and climate + soil + HII modeling frameworks. The same retained predictor sets were applied consistently across all five modeling algorithms.

Following this screening process, a total of 15 environmental variables were retained for modeling the global distribution of *O. stricta* (Table 1). The selected climatic variables included the annual mean temperature (Bio1), mean diurnal temperature range (Bio2), isothermality (Bio3), annual precipitation (Bio12), precipitation of the wettest month (Bio13), and precipitation of the driest month (Bio14). From the soil dataset, six key variables were chosen: AWch, CEC, CLYPPT, CRFVOL, ORCDRC and PHIHOX. In addition, the AI, PET, and HII were incorporated to capture climatic stress, water balance, and anthropogenic impacts. Together, these variables represent the climatic, edaphic, and anthropogenic predictors retained for evaluating the global habitat suitability of *O. stricta*.

### 2.3. Modeling Framework of O. stricta

To compare the predictive performance of different species distribution modeling (SDM) algorithms, five widely used models were selected because of their widespread application in ecological modelling: ANN, RF, GAM, BRT, and MaxEnt. The MaxEnt model was implemented using the standalone MaxEnt software v. 3.4.4 (http://biodiversityinformatics.amnh.org/open_source/maxent/, accessed on 7 July 2025), using default MaxEnt parameters. ANN, RF, GAM, and BRT were implemented in Python (version 3.13). ANN, RF, and BRT were developed using the scikit-learn library (version 1.7.2), while GAM was implemented using the pyGAM library (version 0.11.0).

A common set of 10,000 background points was generated by randomly sampling valid terrestrial grid cells across the calibration area after excluding cells containing missing (NoData) values. The entire terrestrial extent represented by the environmental predictor layers was considered the calibration area for background sampling. No sampling-bias surface or target-group background approach was applied. The same background dataset, occurrence records, and environmental predictor variables were used for all five modeling algorithms to ensure methodological consistency and facilitate direct model comparison [52]. Occurrence records were randomly partitioned into 75% training and 25% testing datasets following established protocols for model validation [34,52]. Predictor variables for the Python-based models were standardized using the training dataset and subsequently applied to the testing dataset.

For the Python-based models, ANN was implemented using an MLPClassifier with three hidden layers (100, 50, and 25 neurons), ReLU activation, early stopping, and a maximum of 1000 iterations. RF was implemented using RandomForestClassifier with 500 trees, a maximum tree depth of 10, a minimum split size of five samples, and a minimum leaf size of two samples. BRT was implemented using GradientBoostingClassifier with 500 boosting iterations, a learning rate of 0.01, a maximum tree depth of five, and a subsampling rate of 0.8. GAM was fitted using LogisticGAM with 10 spline basis functions for each predictor and a maximum of 200 iterations. No additional hyperparameter optimization was performed beyond these predefined parameter settings, and all algorithms were implemented using the same training and testing datasets to ensure consistency in model comparison.

**Figure 2:**
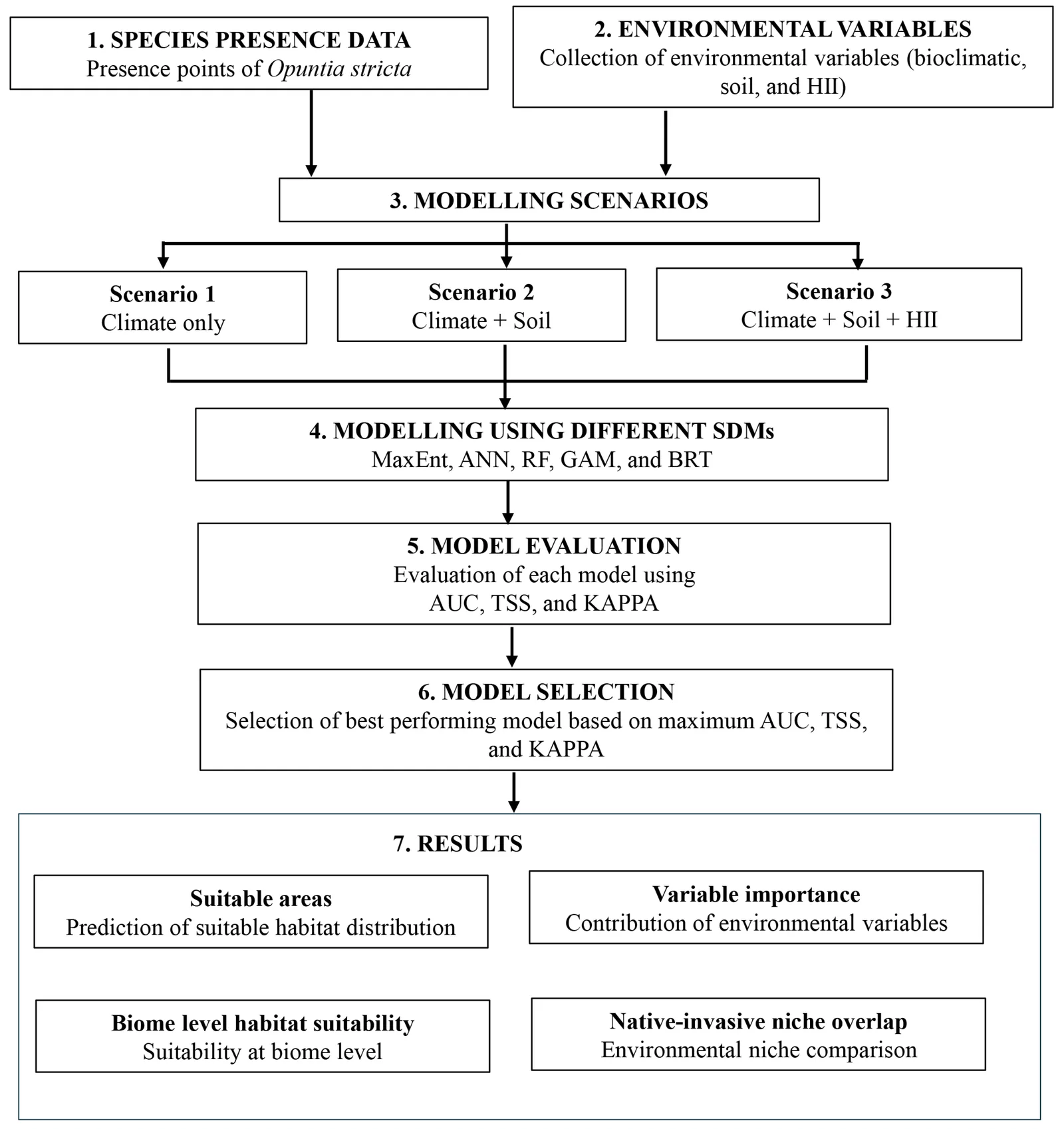
Flowchart showing the integrated workflow for data preparation, modeling, and analysis for *O. stricta.*

Three modelling scenarios were developed to evaluate the influence of different predictor groups: (1) climate only, (2) climate + soil, and (3) climate + soil + human influence. For the global habitat-suitability models, native- and introduced-range occurrence records were pooled to characterize the full range of environmental conditions associated with the current global realized distribution of *O. stricta*. Potential differences between the environmental niches occupied in the native and introduced ranges were evaluated separately using the PCA-env analysis described in Section 2.5. A flowchart summarizing the overall research methodology is presented in Figure 2.

### 2.4. Model Performance Assessment and Validation

Model predictive performance was evaluated using the area under the receiver operating characteristic curve (AUC), true skill statistic (TSS), and Cohen’s kappa. These metrics were used to assess model discrimination and classification performance, whereas probability calibration was not evaluated separately in the present analysis. For each of the 100 model runs, occurrence records were resampled with replacement and partitioned into training and testing subsets using the same procedure across all five algorithms. The same background dataset and predictor layers were retained across replicates to ensure consistent comparison among algorithms. Performance metrics were calculated for each replicate and summarized as mean ± standard deviation. Mean performance values were then compared among the five modeling algorithms, and the model with the strongest overall predictive performance was selected for subsequent habitat-suitability projections. Model evaluation was conducted using the testing datasets in Python (version 3.13) with the scikit-learn library.

The AUC was used to evaluate model discrimination across all probability thresholds, with values ranging from 0.5, indicating performance equivalent to random prediction, to 1.0, indicating perfect discrimination. Values greater than 0.7 were considered to indicate acceptable to good predictive performance [52,53]. TSS was used as a threshold-dependent metric that integrates sensitivity and specificity while remaining independent of species prevalence [54]. TSS values range from −1 to +1, with values approaching +1 indicating stronger classification performance. Cohen’s kappa was used to quantify agreement between predicted and observed classifications while accounting for agreement expected by chance [55]. Kappa values also range from −1 to +1, with higher values indicating stronger agreement.

For binary classification, continuous model predictions were converted into suitable and unsuitable classes using the maximum training sensitivity plus specificity threshold. The resulting binary suitability maps were then used to calculate the proportion of global land area classified as suitable and to quantify suitable area within each biome. For the selected MaxEnt model, the corresponding threshold was applied to the cloglog outputs to generate the final binary habitat-suitability maps. These binary maps were subsequently used to estimate the potential global habitat suitability of *O. stricta*. The final suitability maps presented in this study represent the averaged predictions across the bootstrap replicates of the selected MaxEnt model.

### 2.5. Niche-Overlap Analysis

Niche overlap between the native and introduced ranges of *O. stricta* was quantified using the PCA-env framework following [56] under three species distribution modeling scenarios: (i) climate only, (ii) climate + soil, and (iii) climate + soil + HII. Environmental variables associated with native and invasive occurrence records were projected into a common environmental space using principal component analysis (PCA). Before PCA, the combined environmental data were standardized to zero mean and unit variance.

Occurrence densities were subsequently estimated using Gaussian kernel density estimation [56]. The environmental background comprised all valid 2.5 arc-minute grid cells within the spatial extent of the environmental raster layers (i.e., the whole study area), with up to 50,000 cells randomly sampled without replacement when necessary.

A common environmental background was used for both the native and introduced ranges, and no species-specific accessible-area (M) restriction was applied. The first two principal components (PC1 and PC2) were retained for niche estimation, and the proportion of environmental variation explained by these axes was calculated separately for each modeling scenario.

Kernel densities were estimated on a common 100 × 100 grid in PC1–PC2 space using Gaussian kernel density estimation with Scott’s bandwidth [56]. Following the PCA-env approach, occurrence densities were corrected for environmental availability using the background density.

Niche overlap was quantified using Schoener’s *D* [57] and Hellinger’s *I* [58] statistics, both ranging from 0 (no overlap) to 1 (complete overlap). To test niche equivalency, occurrence points were randomly permuted between ranges and overlap metrics recalculated across 1000 iterations [58]. A niche similarity test was conducted by randomly shifting each range in environmental space 1000 times to assess whether observed overlap exceeded random expectation [56,58]. The similarity test was conducted bidirectionally by separately shifting the native and introduced occurrence distributions against the observed distribution of the other range.

Niche space was partitioned into three components: shared niche (overlap), novel environmental conditions occupied only in the introduced range (niche expansion), and native niche conditions not occupied in the introduced range (unfilled niche) [59].

All analyses were conducted separately for each modeling scenario using Python, with raster processing performed using rasterio, principal component analysis using scikit-learn (http://scikit-learn.org, accessed on 27 July 2026), and kernel density estimation using SciPy (https://scipy.org, accessed on 27 July 2026).

## 3. Results

### 3.1. Temporal Patterns in Reported Occurrence Records

**Figure 3:**
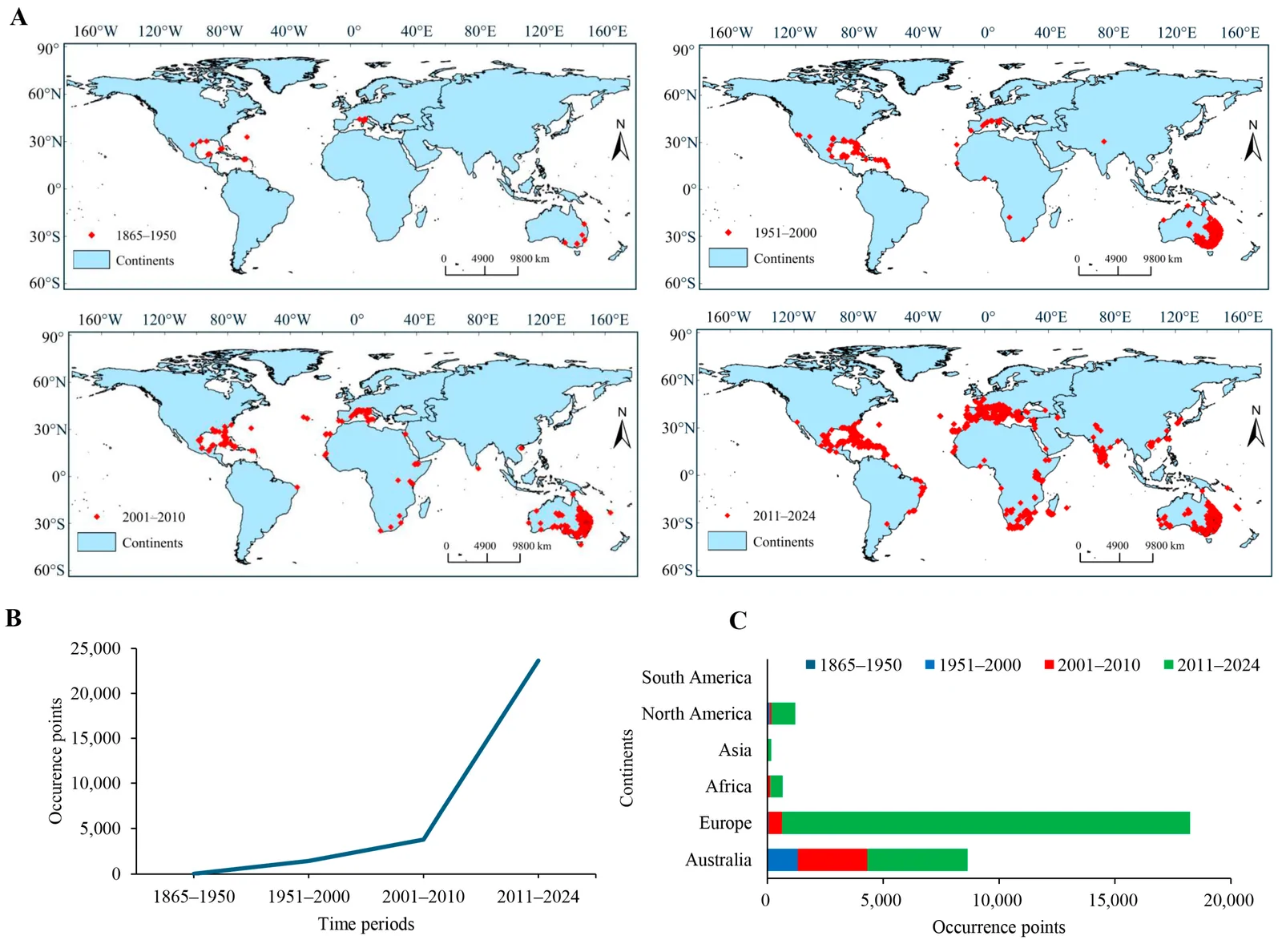
Temporal patterns in reported occurrence records of *O. stricta* from 1865 to 2024. (**A**) Geographic distribution of reported occurrence records across four time periods (1865–1950, 1951–2000, 2001–2010, and 2011–2024). (**B**) Temporal distribution of reported occurrence records. (**C**) Continental distribution of reported occurrence records.

Reported occurrence records of *O. stricta* showed clear temporal and spatial variation during the study period (Figure 3A). From 1865 to 1950, records were limited to scattered locations in the Americas, Africa, and Europe. Between 1951 and 2000, reported occurrences became more widespread, with additional records from Asia and Oceania. The period 2001–2010 showed a further increase in the geographic distribution of reported records, whereas 2011–2024 contained the greatest number of reported occurrences across all continents.

The temporal distribution of reported occurrence records remained relatively low before 2000 but increased markedly thereafter (Figure 3B). The highest number of reported records was observed during 2011–2024, increasing from approximately 5000 to more than 15,000 records.

Continental analysis showed substantial geographic variation in reported occurrence records (Figure 3C). Europe contained the greatest number of records (17,601), followed by Australia (4321), whereas Asia, Africa, and North America had moderate numbers of records, and South America had comparatively fewer records. These results describe temporal and geographic patterns in reported occurrence records.

### 3.2. Model Performance and Selection of a Suitable Model

**Figure 4:**
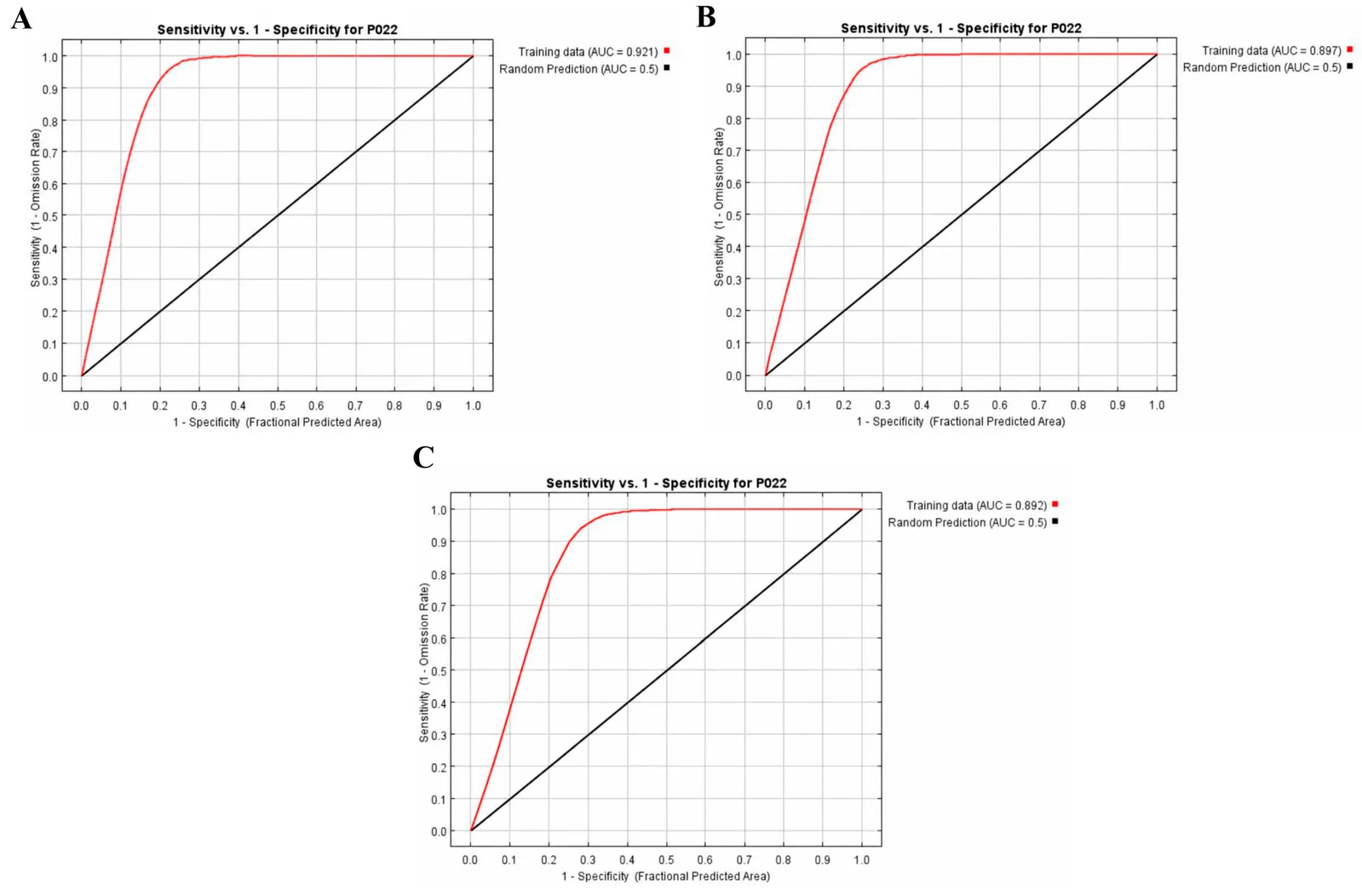
ROC curves showing the predictive performance of MaxEnt model under three environmental variable frameworks. (**A**) Climate-only model; (**B**) climate + soil model; and (**C**) climate + soil + human-influence model.

We compared the predictive performance of five species distribution models (ANN, RF, GAM, MaxEnt, and BRT) across three environmental variable frameworks: climate only, climate + soil, and climate + soil + human (Table 2). Among the evaluated algorithms, MaxEnt showed the strongest overall performance across the combined evaluation metrics and was therefore selected for subsequent habitat-suitability analyses. In the climate-only scenario, MaxEnt achieved an AUC of 0.92 ± 0.01, TSS of 0.89 ± 0.02 and a kappa of 0.89 ± 0.01, indicating excellent discrimination and agreement. With the addition of soil variables, MaxEnt performance remained high, with an AUC of 0.90 ± 0.01, TSS of 0.89 ± 0.02, and kappa of 0.87 ± 0.01. When human variables were further included, performance remained high but declined slightly, with an AUC of 0.89 ± 0.01, TSS of 0.88 ± 0.02, and kappa of 0.84 ± 0.01. In contrast, the other models showed either lower overall accuracy or greater sensitivity to changes in variable sets. These results indicate that MaxEnt showed the strongest overall performance among the evaluated algorithms under the present validation framework and support its selection for subsequent habitat-suitability projections. The ROC curves further indicated high discriminatory ability of the MaxEnt model across all three environmental frameworks under the present validation procedure (Figure 4).

### 3.3. Contribution of Environmental Variables to O. stricta Distribution

Variable importance analysis revealed distinct contributions across the three modeling frameworks (Table 3). In the climate-only model, climatic variables had a substantial influence, with PET contributing the greatest percentage (35.75%), followed by Bio14 (27.75%) and Bio3 (17.46%). Permutation importance similarly demonstrated the dominance of Bio1 (32.73%), AI (24.53%), and Bio14 (13.06%).

In the climate + soil model, climatic variables maintained their predominant role, with Bio14 (29.58%), Bio3 (25.09%), PET (14.96%), and Bio1 (8.20%) contributing the most. Among the soil properties, PHIHOX was notably important (13.37%), whereas the other soil variables, such as CLYPPT (0.50%) and CEC (0.31%), contributed minimally. Permutation importance reinforced the primacy of climatic factors, with Bio1 (35.01%), AI (20.56%), and Bio14 (12.48%) ranking highest.

Similarly, the climate + soil + human model exhibited continued climatic dominance, with Bio14 (30.72%), Bio3 (26.65%), and Bio1 (9.34%) contributing most substantially. Soil variables, particularly PHIHOX (7.23%), had a moderate influence, whereas CEC (0.19%) remained minor. Notably, the HII contributed only 0.81% to the climate + soil + human model, representing the lowest contribution among the environmental variables considered. Permutation importance consistently emphasized climatic variables, with Bio1 (28.34%), AI (20.05%), and Bio14 (19.58%) as primary predictors.

Overall, Climatic variables provided the strongest predictive information for *O. stricta* habitat suitability, whereas soil variables provided secondary information and HII contributed comparatively little additional predictive information within the evaluated framework. These findings indicate that climatic conditions were the dominant predictors of habitat suitability for *O. stricta* in this study.

### 3.4. Predicted Global Habitat Suitability Across Modeling Frameworks

**Figure 5:**
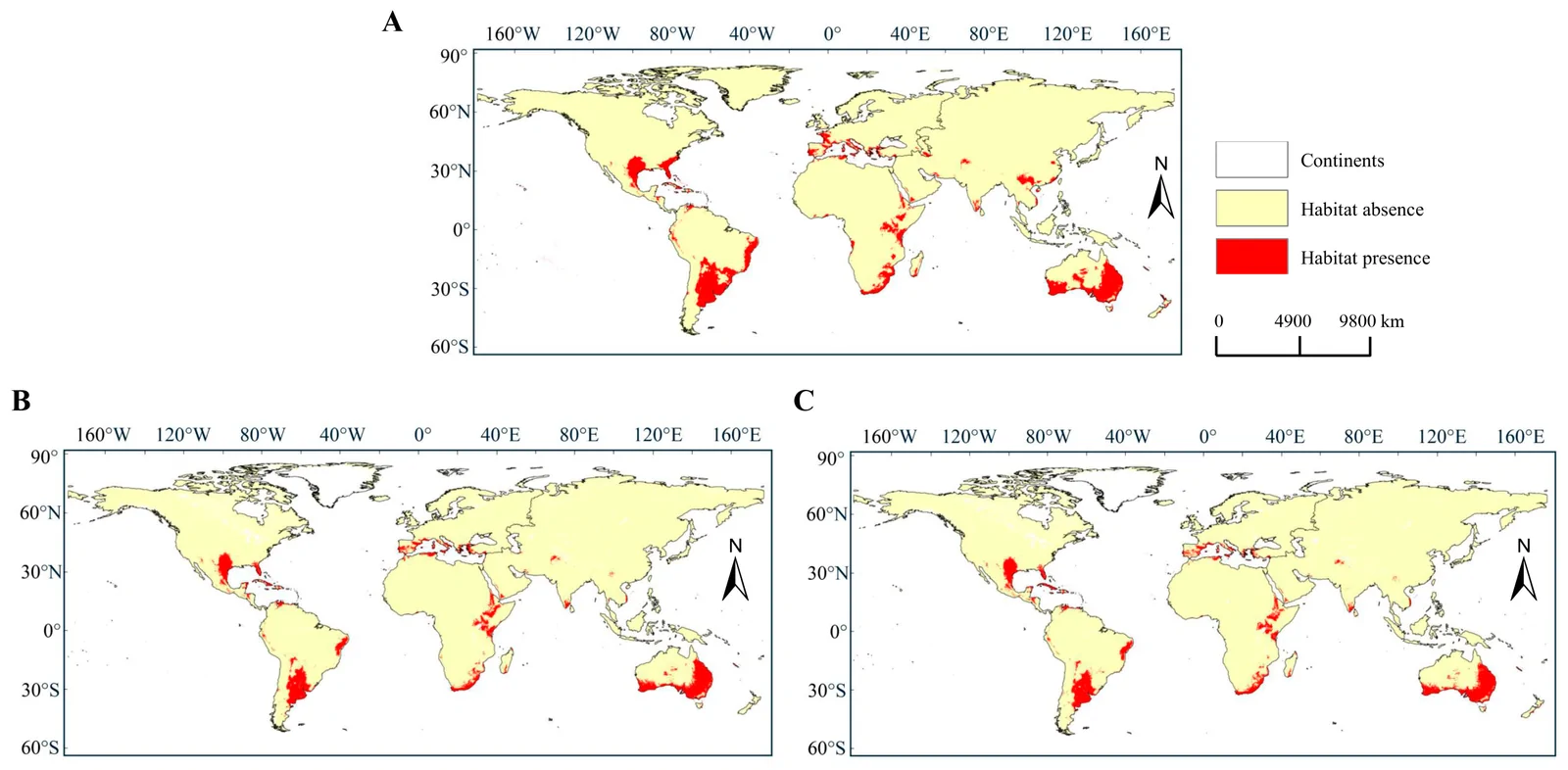
Potential global habitat distribution of *O. stricta* predicted using three modeling frameworks. (**A**) Predicted suitable habitat based on climate-only variables; (**B**) predicted suitable habitat based on climate and soil variables (climate + soil). (**C**) Predicted suitable habitat based on climate, soil, and human-influence variables (climate + soil + human). Red areas indicate predicted habitat presence, whereas white areas indicate habitat absence worldwide.

The predicted global habitat suitability of *O. stricta* varied markedly across the three modeling frameworks (Figure 5A–C). The climate-only model predicted the largest extent of suitable habitat, whereas the inclusion of soil variables reduced the extent of areas classified as suitable. Incorporating the HII produced a further, although relatively small, reduction in predicted suitable area. These differences indicate that additional environmental predictors refine habitat-suitability estimates beyond climate alone rather than demonstrating that soil or human influence independently limits the species’ distribution.

**Figure 6:**
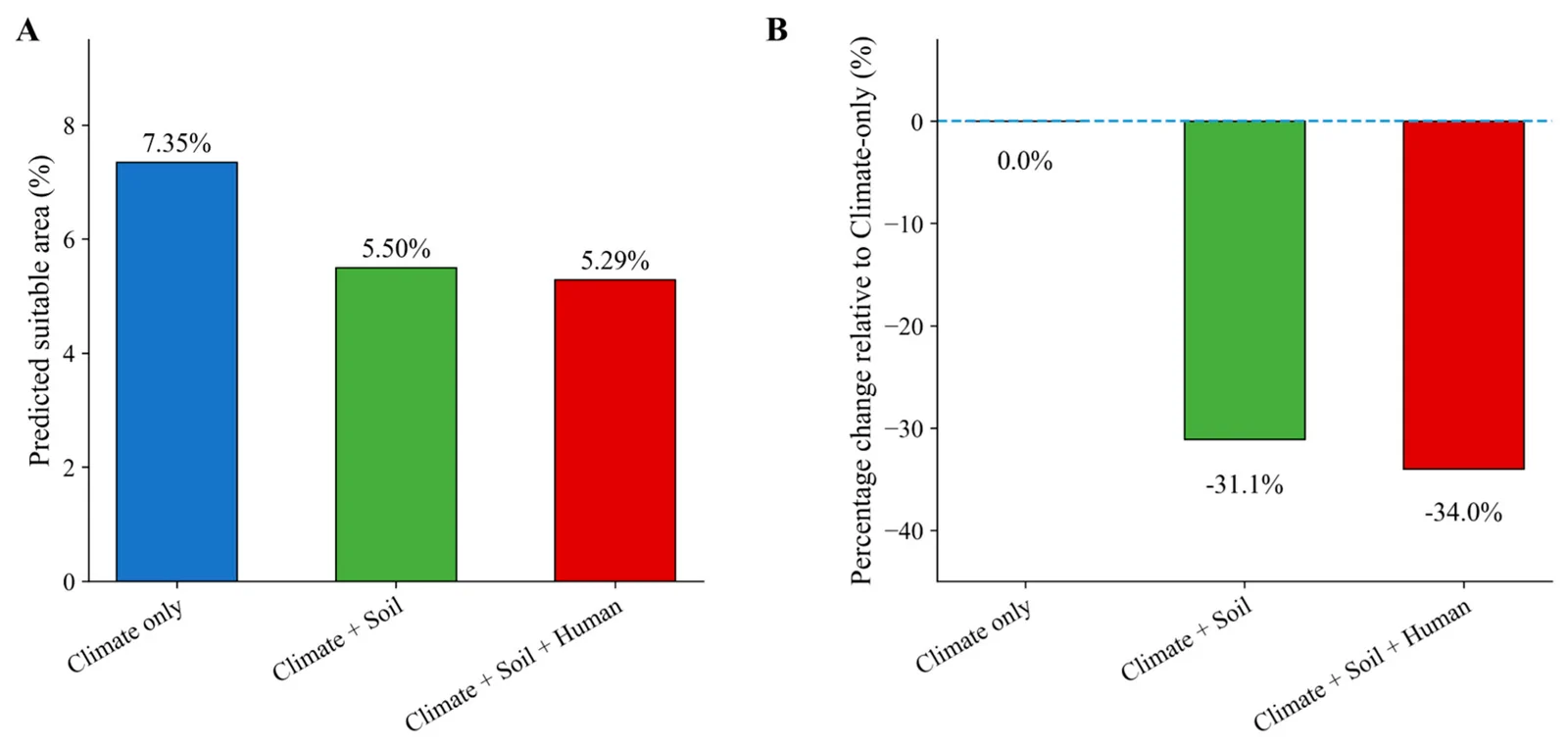
Predicted suitable habitat area and relative change across model complexity scenarios. (**A**) Predicted suitable area (%) estimated by three model types: climate only, climate + soil, and climate + soil + human. (**B**) Percentage change in the predicted suitable area relative to the climate-only model.

Quantitatively, the proportion of global land area classified as suitable decreased from 7.35% in the climate-only model to 5.50% in the climate + soil model and further to 5.29% in the climate + soil + human model (Figure 6A). Relative to the climate-only baseline, the suitable area decreased by 31.09% when soil variables were included and by 34.04% when both soil and human influence were incorporated (Figure 6B). Overall, the predicted extent of suitable habitat varied among the three modeling frameworks, with progressively smaller suitable areas predicted as additional environmental variables were incorporated.

### 3.5. Native-Introduced Niche Overlap Across Three Modeling Scenarios

Native-introduced niche overlap differed among the three species distribution modeling scenarios (Table 4). Under the climate-only scenario, Schoener’s *D* and Hellinger’s *I* were 0.60 and 0.76, respectively, indicating moderate overlap between the native and invasive environmental niches. Niche stability was estimated at 0.71, whereas expansion and unfilling were 0.29 and 0.13, respectively.

The inclusion of soil variables increased niche overlap, with Schoener’s *D* and Hellinger’s *I* increasing to 0.65 and 0.86, respectively. Niche stability also increased to 0.87, while niche expansion declined to 0.13. Unfilling increased slightly to 0.16. Following the incorporation of HII, Schoener’s *D* and Hellinger’s *I* increased marginally to 0.68 and 0.87, respectively. Niche stability increased to 0.91, whereas niche expansion decreased slightly to 0.09, with unfilling remaining constant at 0.16. Niche equivalency and similarity tests were significant across all three modeling scenarios (*p* < 0.05). The significant equivalency test results indicated that the native and invasive niches were not statistically equivalent, whereas the significant similarity test results indicated that the observed niche overlap was significantly different from that expected under the respective null hypothesis. Collectively, these results indicate that the incorporation of additional environmental predictors was associated with progressively greater native-introduced niche overlap, increased niche stability, and reduced niche expansion.

### 3.6. Predicted Habitat Suitability of O. stricta Across Different Biomes

The model predicted substantial variation in habitat suitability for *O. stricta* across different global biomes (Table 5). Mediterranean forests, woodlands, and scrublands had the highest proportion of biome area predicted as suitable, with 89,923 suitable cells representing 48.47% of the biome area. In contrast, tropical and subtropical grasslands, savannas, and shrublands contained the greatest absolute number of suitable cells (246,165), although these represented a lower proportion of the biome area (25.17%). Tropical and subtropical coniferous forests had 5647 suitable cells, representing 15.68% of their biome area, followed by temperate grasslands, savannas, and shrublands with 97,082 suitable cells (14.55%), and mangroves with 2402 cells (14.44%).

Among the remaining biomes, tropical and subtropical dry broadleaf forests had 15,814 suitable cells (10.69%), while tropical and subtropical moist broadleaf forests had 96,135 suitable cells (10.08%). Temperate coniferous forests and temperate broadleaf and mixed forests contained 21,949 (8.03%) and 60,744 (7.18%) suitable cells, respectively. Flooded grasslands and savannas contained 7109 suitable cells (12.26%), whereas lakes, montane grasslands and shrublands, and deserts and xeric shrublands contained 5778 (8.45%), 16,485 (5.73%), and 84,922 (5.61%) suitable cells, respectively.

Boreal forests/taiga, tundra, and rock and ice showed no suitable cells, indicating climatic thresholds beyond the species’ tolerance range. These patterns demonstrate that the environmental suitability of *O. stricta* is concentrated in Mediterranean climate regions, subtropical grasslands, and warm temperate ecosystems, while cold-climate biomes remain unsuitable.

## 4. Discussion

*O. stricta* poses complex ecological and socioeconomic challenges at broad geographic scales, requiring integrative, spatially explicit assessments to guide prevention and management. By applying a hierarchical modeling framework that sequentially incorporated climatic, edaphic, and anthropogenic layers, this study identified the relative importance of environmental predictors associated with global habitat suitability and produced habitat-suitability maps that can support surveillance and management. Our approach provides a correlative assessment of species–environment relationships rather than solely reporting area statistics, thereby improving our understanding of the environmental conditions associated with habitat suitability for *O. stricta*. Together, these results allow us to synthesize the ecological mechanisms, biogeographic patterns, and management implications that emerge from these analyses.

Among the evaluated SDMs, MaxEnt showed the strongest overall predictive performance across the three environmental frameworks under the present validation procedure. However, because model evaluation was based on randomly partitioned training and testing data, these performance estimates should be interpreted cautiously, as residual spatial dependence between geographically proximate occurrence records may have contributed to optimistic estimates of model discrimination. This finding is consistent with those of previous studies showing that MaxEnt performs well for modeling presence-only data and invasive species [33,34,60]. Model complexity can influence the balance between model fit and transferability, with overly complex models potentially leading to overfitting by capturing relationships that are specific to the calibration dataset rather than broadly transferable [61]. The relatively consistent performance of MaxEnt across the three predictor frameworks supports its use for subsequent interpretation of environmental correlates of *O. stricta* habitat suitability within the present modeling framework.

The variable importance analysis identified Bio14 and PET as major predictors of habitat suitability for *O. stricta*. These results indicate that moisture-related climatic variables are strongly associated with the predicted distribution of the species. Previous physiological studies have suggested that water availability during dry periods and atmospheric evaporative demand influence the performance of *Opuntia* species under arid condition [62,63]. Although these physiological responses were not evaluated directly in the present study, the strong contributions of Bio14 and PET are consistent with their proposed role in shaping the environmental conditions associated with habitat suitability. Temperature-related variables also contributed substantially to model performance. Experimental evidence indicates that CAM photosynthesis in *Opuntia* species is most efficient within moderate day and night temperature ranges, whereas prolonged exposure to freezing temperatures reduces growth and survival [62,64]. These physiological characteristics are consistent with the predicted absence of suitable habitat in boreal and tundra biomes, although the present correlative modeling framework does not directly evaluate the underlying mechanisms. Collectively, our findings are consistent with previous studies identifying climatic conditions as the primary environmental correlates of *Opuntia* distributions across arid and semiarid ecosystems [26].

The sequential incorporation of environmental layers revealed progressive refinement of suitable habitat predictions. The climate-only model predicted 7.35% of the global land area as suitable, which decreased to 5.50% with soil variables and further to 5.29% when the HII was included, representing cumulative reductions of 31.1% and 34.0%, respectively (Figure 5). These contractions suggest that the inclusion of soil variables and HII refined the prediction of climatically suitable habitats, although climatic variables remained the dominant predictors of habitat suitability. Among the edaphic variables, soil pH had the highest contribution (7.23%), which is consistent with observations that *Opuntia* species tolerate broad pH ranges (4.5–8.5) compared with those of their native competitors [65]. The relatively minor contributions of clay content and cation exchange capacity suggest that these soil properties exert a comparatively limited influence on the predicted habitat suitability of *O. stricta*. This finding is consistent with previous studies reporting that *O. stricta* can establish across a wide range of soil conditions, including nutrient-poor and disturbed substrates [66].

Although HII contributed only 0.81% to the final MaxEnt model, this finding should be interpreted cautiously. HII is a broad composite indicator reflecting human population density, land use, accessibility, and infrastructure, but it does not explicitly represent important invasion pathways such as ornamental planting, intentional cultivation, livestock movement, road-mediated dispersal, horticultural trade, or propagule pressure [12,47]. Moreover, the occurrence records used in this study reflect a long history of human-assisted introductions, meaning that part of the anthropogenic signal may already be embedded within the observed distribution or overlap with climatic and environmental predictors [5,10]. Consequently, the low contribution of HII indicates that this variable provided limited additional predictive information beyond the climatic and edaphic variables included in the model, rather than demonstrating that human activities play a negligible role in the invasion of *O. stricta*. This interpretation is consistent with previous studies showing that anthropogenic activities are often more influential in species introduction and dispersal than in determining local environmental suitability [5,12,67].

The pooled global SDM and the native-introduced niche analysis address complementary but distinct questions. The pooled model characterizes environmental habitat suitability across the range of conditions currently occupied by *O. stricta* worldwide, whereas the PCA-env analysis evaluates whether the environmental space occupied in the introduced range differs from that occupied in the native range. Therefore, the global suitability maps should be interpreted as estimates of environmental habitat suitability rather than as formal predictions of introduction probability or complete invasion risk outside the native range. Within this complementary framework, the niche-overlap analysis further demonstrated that incorporating additional environmental predictors progressively refined the environmental niche of *O. stricta*. Compared with the climate-only analysis, the inclusion of soil variables was associated with greater native-introduced niche overlap and stability and with lower estimated niche expansion. The subsequent inclusion of HII produced comparatively smaller changes, suggesting that HII provided limited additional differentiation of the environmentally suitable niche. These findings are consistent with previous studies showing that integrating environmental dimensions beyond climate can improve characterization of species niches [56,68]. Nevertheless, because the present analyses are based on correlative niche models, the observed patterns should be interpreted as differences in occupied environmental space rather than as direct evidence of ecological or evolutionary niche shifts [59]. Together, these findings highlight the value of incorporating complementary environmental predictors when characterizing native-introduced niche relationships and predicting habitat suitability for *O. stricta*.

The biome-level assessment was based on the final integrated model, providing ecologically refined estimates of habitat suitability after accounting for climatic, soil, and anthropogenic environmental filters. Mediterranean forests, woodlands, and scrublands showed the highest proportional habitat suitability for *O. stricta*, with 48.47% of the biome area classified as suitable. In contrast, tropical and subtropical grasslands, savannas, and shrublands contained the greatest absolute number of suitable cells because of their larger geographic extent, despite having lower proportional suitability (25.17%). These metrics represent different aspects of predicted habitat suitability: proportional suitability indicates the fraction of a biome classified as suitable, whereas the absolute number of suitable cells reflects the total extent of predicted suitable habitat within that biome. Importantly, neither metric alone determines management priority, which also depends on factors such as current invasion status, propagule pressure, ecological impacts, accessibility, and feasibility of control [67,68].

The high proportional suitability observed in Mediterranean regions is consistent with the documented occurrence of *O. stricta* under warm, seasonally dry environmental conditions [19,26]. However, the present correlative analysis does not directly test the physiological mechanisms underlying this association. Tropical and subtropical grasslands may provide favorable conditions for establishment, particularly in areas subject to disturbances such as grazing and periodic fire. Previous studies have suggested that grazing, fire, and other disturbances can facilitate the establishment and persistence of *O. stricta* in grassland systems [11]. Because these mechanisms were not evaluated directly in the present study, they should be regarded as possible explanations for the modeled suitability patterns rather than as mechanisms demonstrated by our analysis. In contrast, deserts and xeric shrublands showed relatively low predicted suitability (5.61%). Previous physiological studies suggest that extreme water limitation can restrict the performance of CAM plants [63], although this mechanism was not tested directly in the present analysis. Similarly, the absence of predicted suitability in boreal forests and tundra is consistent with previous evidence that low temperatures and freezing conditions can limit the performance of *Opuntia* species [64].

The marked increase in reported occurrence records after 2000 likely reflects a combination of biological and observational factors (Figure 3). *O. stricta* possesses several traits known from previous studies to facilitate establishment and dispersal, including vegetative reproduction through detached cladodes and seed dispersal by vertebrates [16,22,69]. Human-mediated pathways, including horticultural introduction and global transport, may also contribute to its broad geographic distribution [10,11]. However, these biological and anthropogenic processes were not directly tested by the temporal occurrence analysis. In addition, the increase in reported records may partly reflect greater biodiversity survey effort, digitization of historical collections, and the expansion of citizen-science platforms such as GBIF. Therefore, the temporal pattern presented here should be interpreted as a change in reported occurrence records rather than as direct evidence of a prolonged lag phase or exponential biological spread.

The present modeling framework is correlative and identifies statistical associations between environmental predictors and the observed distribution of *O. stricta*. Biological mechanisms proposed in previous studies, including physiological responses to temperature and moisture, CAM-related adaptations, soil tolerance, disturbance responses, and dispersal processes, may help explain some of the modeled patterns but were not directly tested in this study. Accordingly, these mechanisms are discussed as literature-supported explanations or hypotheses rather than as causal processes demonstrated by the present analysis.

The management implications of *O. stricta* invasion are complicated by conflicting ecological and economic considerations. The species has been cultivated for centuries as livestock fodder in arid regions where forage alternatives are limited, with cladodes containing high moisture content (80–90%) and moderate protein levels (4–8% dry weight) [70,71,72]. In Mexico, Central America, and Mediterranean countries, *Opuntia* cultivation supports significant agricultural industries that produce cladodes (nopalitos), fruits (tunas), and cochineal dye [73]. The fruits are consumed by numerous bird and mammal species, potentially providing food resources [69]. The species also exhibits potential for biofuel production and carbon sequestration [74]. These beneficial uses create complex socioecological trade-offs, particularly where invasions intersect with subsistence agriculture and pastoralism [26]. Nevertheless, the substantial ecological and economic damage documented in invaded regions necessitate robust management strategies.

Effective management requires integrated approaches that combine prevention, early detection, and control measures. Prevention through strengthened biosecurity and horticultural trade regulation remains the most cost-effective strategy for limiting new introductions [1]. Early detection and rapid response targeting small, emerging populations significantly increase eradication success rates [75]. With respect to established infestations, biological control has produced the most substantial long-term outcomes, exemplified by Cactoblastis cactorum in Australia, which reduced millions of hectares of infestation within a decade, clearing 80% of Queensland’s infested land and 50–60% of New South Wales’ land by 1933 [76]. However, the nontargeted impacts observed in North America underscore the necessity of rigorous host-specificity testing [21,77]. More targeted agents, such as *Dactylopius* species, have achieved effective suppression in South Africa, particularly in arid regions [78]. Mechanical and chemical control can supplement biological approaches but typically requires sustained effort and repeated applications [79].

Several limitations should be acknowledged when interpreting these results. The occurrence records used in this study are subject to temporal and spatial variation in sampling effort and reporting intensity. Therefore, temporal patterns should be interpreted as trends in reported occurrences rather than as direct evidence of invasion dynamics. The human influence index represents conditions from 1995 to 2004 and may not fully capture more recent anthropogenic changes, such as intensified land-use conversion or shifts in population density. In addition, the 2.5 arc-minute spatial resolution, while appropriate for a global assessment, may overlook fine-scale environmental variability, particularly in fragmented landscapes and island ecosystems. Although occurrence records were spatially rarefied before modeling, model evaluation relied on random training–testing partitions rather than geographically independent spatial cross-validation. Residual spatial dependence between training and testing records may therefore have inflated estimates of model performance. Future studies should compare random partitioning with spatial block, environmental block, or region-based cross-validation to more rigorously evaluate model transferability across geographic regions. The present assessment also focused on current climatic suitability and did not incorporate future climate scenarios, limiting our ability to anticipate distributional changes under projected environmental change. Future research should therefore incorporate updated human-influence datasets, higher-resolution environmental layers, geographically independent validation approaches, and projections from multiple climate models to strengthen prediction robustness and support more effective early detection, monitoring, and management under current and future environmental conditions.

Our results identify substantial current habitat suitability for *O. stricta* in Mediterranean, subtropical, and warm-temperate regions. The marked increase in reported occurrences in recent decades, together with the species’ documented ecological impacts, supports continued surveillance and early detection. Management priorities should integrate habitat-suitability information with current occupancy, propagule pressure, ecological impacts, and control feasibility. Strengthened biosecurity, early detection, rapid response, and appropriate biological-control strategies may help reduce impacts in regions where the species is established or likely to encounter favorable environmental conditions.

## 5. Conclusions

This study presents a comprehensive 160-year assessment of temporal and geographic patterns in reported occurrences of *O. stricta*, with the greatest increase in reported records observed during the 21st century. Among the evaluated species distribution models, MaxEnt showed the strongest overall performance across the combined AUC, TSS, and kappa metrics and was therefore selected for subsequent habitat-suitability analyses. Climatic variables, particularly precipitation of the driest month, isothermality, and potential evapotranspiration, were the strongest predictors of global habitat suitability, whereas edaphic variables provided secondary predictive information and the human influence index contributed comparatively little additional information within the evaluated modeling framework.

The hierarchical multivariable modeling framework showed that incorporating edaphic and anthropogenic predictors progressively refined estimates of environmental habitat suitability relative to the climate-only model. Similarly, native-introduced niche analyses indicated that the inclusion of soil and human-influence variables was associated with greater niche overlap and stability and lower estimated niche expansion. These patterns emphasize the value of incorporating multiple environmental dimensions when characterizing the environmental conditions associated with the global distribution of *O. stricta*. At the biome level, Mediterranean forests, woodlands, and scrublands showed the highest proportional habitat suitability, whereas tropical and subtropical grasslands, savannas, and shrublands contained the greatest absolute number of suitable cells. Boreal and tundra regions showed little or no predicted suitability, consistent with the species’ known sensitivity to cold environmental conditions.

The resulting habitat-suitability patterns can support surveillance, early detection, and management planning by identifying regions where environmental conditions are favorable for *O. stricta*. However, these suitability estimates should not be interpreted as complete measures of invasion probability, which also depends on propagule pressure, introduction pathways, dispersal, disturbance, and management conditions. Beyond *O. stricta*, the integrated framework combining temporal occurrence-pattern assessment, multivariable species distribution modeling, native-introduced niche analysis, and biome-level habitat-suitability assessment provides a useful approach for evaluating the environmental suitability and niche relationships of invasive species at broad geographic scales.

## Figures and Tables

**Table 1:** List of selected environmental variables for modeling the suitable potential habitat of *O. stricta.* All the original resolution datasets are resampled to a 2.5 arc-minute resolution.

Variables	Description	Unit	Original Resolution	Source
AI	Aridity index	–	30 arc-sec	CGIAR-CSI Global database
PET	Potential evapotranspiration	mm	30 arc-sec	CGIAR-CSI Global database
Bio1	Annual mean temperature	°C	2.5 arc-min	www.paleoclim.org
Bio2	Mean diurnal temperature range	°C	2.5 arc-min	www.paleoclim.org
Bio3	Isothermality (Bio2/Bio7) (×100)	–	2.5 arc-min	www.paleoclim.org
Bio12	Annual precipitation	mm	2.5 arc-min	www.paleoclim.org
Bio13	Precipitation of wettest month	mm	2.5 arc-min	www.paleoclim.org
Bio14	Precipitation of driest month	mm	2.5 arc-min	www.paleoclim.org
AWch	Available soil water capacity	–	30 arc-sec	ISRIC-World Soil Information
CEC	Cation exchange capacity	cmolc/kg	30 arc-sec	ISRIC-World Soil Information
CLYPPT	Soil texture fraction clay in percent	–	30 arc-sec	ISRIC-World Soil Information
CRFVOL	Coarse fragments volumetric in percent	–	30 arc-sec	ISRIC-World Soil Information
ORCDRC	Soil organic carbon content in g per kg	–	30 arc-sec	ISRIC-World Soil Information
PHIHOX	Soil pH × 10 in H_2_O	–	30 arc-sec	ISRIC-World Soil Information
HII	Human influence index	–	30 arc-sec	Socioeconomic Data and Application center (SEDAC)

**Table 2:** Comparison of the predictive performance (mean ± SD) of five species distribution models (SDMs) under three environmental predictor frameworks: climate only, climate + soil, and climate + soil + human variables.

Model ^a^	Climate Only			Climate + Soil			Climate +Soil + Human		
	**AUC**	**TSS**	**Kappa**	**AUC**	**TSS**	**Kappa**	**AUC**	**TSS**	**Kappa**
ANN	0.91 ± 0.01	0.81 ± 0.03	0.80 ± 0.02	0.89 ± 0.02	0.84 ± 0.02	0.83 ± 0.03	0.89 ± 0.02	0.82 ± 0.03	0.81 ± 0.02
RF	0.91 ± 0.01	0.86 ± 0.02	0.85 ± 0.04	0.90 ± 0.02	0.87 ± 0.03	0.86 ± 0.02	0.87 ± 0.04	0.86 ± 0.02	0.85 ± 0.02
GAM	0.89 ± 0.02	0.78 ± 0.03	0.77 ± 0.03	0.87 ± 0.02	0.80 ± 0.03	0.79 ± 0.03	0.87 ± 0.02	0.79 ± 0.03	0.78 ± 0.03
**MaxEnt**	**0.92 ± 0.01**	**0.89 ± 0.02**	**0.89 ± 0.01**	**0.90 ± 0.01**	**0.89 ± 0.02**	**0.87 ± 0.01**	**0.89 ± 0.01**	**0.88 ± 0.02**	**0.84 ± 0.01**
BRT	0.88 ± 0.02	0.85 ± 0.02	0.84 ± 0.04	0.89 ± 0.02	0.87 ± 0.02	0.86 ± 0.02	0.89 ± 0.02	0.85 ± 0.04	0.84 ± 0.02

^a^ Performance comparison of five species distribution models: ANN (artificial neural network), RF (random forest), GAM (generalized additive model), MaxEnt (maximum entropy), and BRT (boosted regression trees) under three environmental variable frameworks (climate only, climate + soil, and climate + soil + human variables). Values are presented as mean ± standard deviation (SD) based on 100 model runs, where the SD quantifies the stability of model performance across repeated model runs. Climatic predictors include Bio1, Bio2, Bio3, Bio12, Bio13 and Bio14, AI (aridity index), and PET (potential evapotranspiration). Soil predictors include AWCh (available water capacity), CEC (cation exchange capacity), CLYPPT (clay content), CRFVOL (coarse fragments), ORCDRC (soil organic carbon), and PHIHOX (soil pH). The human predictor included is the HII (human influence index). Bold values highlight the highest performing model within each variable framework based on the AUC, TSS, and kappa values.

**Table 3:** Percent contribution and permutation importance of environmental variables in the climate-only, climate + soil, and climate + soil + human models for predicting *O. stricta* habitat suitability.

Variables ^a^	Climate Only		Climate + Soil		Climate + Soil + Human	
	**Percent Contribution**	**Permutation**	**Percent Contribution**	**Permutation Imp**	**Percent Contribution**	**Permutation Imp**
AI	1.78	**24.53**	2.31	**20.56**	1.43	**20.05**
PET	**35.75**	**6.67**	**14.96**	**8.62**	**18.33**	**6.56**
Bio1	8.80	**32.73**	**8.20**	**35.01**	**9.34**	**28.34**
Bio2	3.51	6.85	2.68	6.26	1.86	4.20
Bio3	**17.46**	**12.22**	**25.09**	**9.55**	**26.65**	**14**
Bio12	5.01	3.87	2.31	4.39	2.76	4.59
Bio13	0.01	0.08	0.33	0.06	0.13	0.09
Bio14	**27.75**	**13.06**	**29.58**	**12.48**	**30.72**	**19.58**
AWch	–	–	0.01	0.08	0.01	0.08
CEC	–	–	0.31	0.31	0.19	0.18
CLYPPT	–	–	0.43	0.50	0.23	0.25
CRFVOL	–	–	0.33	0.20	0.25	0.17
ORCDRC	–	–	0.09	0.41	0.04	0.24
PHIHOX	–	–	**13.37**	1.54	**7.23**	0.78
HII	–	–	–	–	0.81	0.87

^a^ AI, aridity index; PET, potential evapotranspiration; Bio1–Bio14, bioclimatic variables; AWCh, available water capacity; CEC, cation exchange capacity; CLYPPT, clay content; CRFVOL, coarse fragments; ORCDRC, soil organic carbon; PHIHOX, soil pH; HII, human influence index. Percent contribution and permutation importance values derived from MaxEnt model outputs. Bold values highlight the dominant predictors. Empty cells (–) indicate variables excluded from the modeling framework.

**Table 4:** Comparison of native and invasive niche dynamics under modelling scenarios.

Scenarios	Schoener’s D	Hellinger’s I	Stability	Expansion	Unfilling	Equivalency Test (p)	Similarity Test (p)
Climate	0.60	0.76	0.71	0.29	0.13	0.04	0.02
Climate + Soil	0.65	0.86	0.87	0.13	0.16	0.02	0.01
Climate + Soil + HII	0.68	0.87	0.91	0.09	0.16	0.02	0.01

Niche overlap and dynamics between the native and invasive ranges of *O. stricta* under three modelling scenarios (climate only; climate + soil; climate + soil + HII). Schoener’s D and Hellinger’s I measure niche overlap (0 = none, 1 = complete). Stability represents the proportion of the introduced niche overlapping with the native niche, expansion represents the proportion of the introduced niche occurring under novel environmental conditions, and unfilling represents the proportion of the native niche that remains unoccupied in the introduced range. Equivalency and Similarity test *p*-values are based on 1000 randomizations; *p* < 0.05 indicates a significant departure from random expectations.

**Table 5:** Biome-level predicted habitat suitability of *O. stricta.*

Biome Types	Count	Cells Covered	Area Covered (%)
Tropical and subtropical moist broadleaf forests	953,018	96,135	10.08
Tropical and subtropical dry broadleaf forests	147,993	15,814	10.69
Tropical and subtropical coniferous forests	36,019	5647	15.68
Temperate broadleaf and mixed forests	845,769	60,744	7.18
Temperate coniferous forests	273,319	21,949	8.03
Boreal forests/taiga	1,429,590	0	0
Tropical and subtropical grasslands, savannas, and shrublands	978,010	246,165	25.17
Temperate grasslands, savannas, and shrublands	667,336	97,082	14.55
Flooded grasslands and savannas	57,984	7109	12.26
Montane grasslands and shrublands	287,606	16,485	5.73
tundra	1,085,189	0	0
Mediterranean forests, woodlands, and scrublands	185,524	89,923	48.47
Deserts and xeric shrublands	1,512,309	84,922	5.61
Mangroves	16,637	2402	14.44
Lakes	68,361	5778	8.45
Rock and ice	329,540	0	0

The table presents modeled suitability estimates for *O. stricta* across different biomes, highlighting extent of predicted habitat suitability based on predicted suitable cell counts and the percentage of the biome area predicted to be environmentally suitable. Values were derived from the final MaxEnt model incorporating climate, soil, and human influence (climate + soil + HII) at a 2.5 arc-minute spatial resolution.

## Data Availability

The raw data supporting the conclusions of this article will be made available by the authors on request.

## Supplementary Materials

The following supporting information can be downloaded at https://www.mdpi.com/article/10.3390/biology15181635/s1. Table S1: List of all environmental variables used in this study; Table S2: Pearson correlation analysis for selecting bioclimatic variables of *O. stricta*.
